# Long-term outcome of adjuvant radiotherapy upon postoperative relapse of centrally located hepatocellular carcinoma: a real-world study

**DOI:** 10.1038/s41598-024-59180-7

**Published:** 2024-04-12

**Authors:** Changcheng Tao, Nan Hu, Yue Liu, Hongwei Wang, Zhihao Wang, Kai Zhang, Liming Wang, Bo Chen, Fan Wu, Weiqi Rong, Jianxiong Wu

**Affiliations:** 1 Department of Hepatobiliary Surgery, National Cancer Center/National Clinical Research Center for Cancer/Cancer Hospital, Chinese Academy of Medical Sciences and Peking Union Medical College, Beijing, 100021 China; 2 Department of Radiation Oncology, National Cancer Center/National Clinical Research Center for Cancer/Cancer Hospital, Chinese Academy of Medical Sciences and Peking Union Medical College, Beijing, 100021 China

**Keywords:** Centrally located hepatocellular carcinoma, Adjuvant radiotherapy, Surgical resection, Relapse, Radiotherapy, Liver cancer

## Abstract

Despite that surgical resection is widely regarded as the most effective approach to the treatment of liver cancer, its safety and efficacy upon centrally located hepatocellular carcinoma (HCC) remain unsatisfactory. In consequence, seeking an integrated treatment, like combined with adjuvant radiotherapy, to enhance the prognosis of patients is of critical importance. By recruiting patients undergoing surgical resection for centrally located HCC ranging from June 2015 to 2020, they were divided into liver resection combined with adjuvant radiotherapy (LR + RT) and mere liver resection (LR) groups. The calculation of propensity score and model of Cox proportional hazards regression were utilized. 193 patients were recruited in aggregation, containing 88 ones undergoing LR + RT, while 105 handled with LR. RT was verified to be an independent factor of prognosis for relapse (HR 0.60). In propensity-score analyses, significant association existed between adjuvant radiotherapy and better disease-free survival (DFS) (Matched, HR 0.60; Adjustment of propensity score, HR 0.60; Inverse probability weighting, HR 0.63). The difference of DFS was apparent within two groups (*p* value = 0.022), and RT significantly down-regulated early relapse (*p* value < 0.05) in subgroup analysis. The calculation of E-value revealed robustness of unmeasured confounding. The combination of liver surgical resection with RT is safe and effective towards patients with centrally located HCC, which would notably enhance the prognosis and decrease the early relapse of HCC.

## Introduction

As the globally fourth-leading factor of cancer-associated death, hepatocellular carcinoma (HCC) takes up almost 90% of primary liver cancer (PLC)^1^, which is a fatal illness accompanied by severe morbidity, negative prognosis, along with a series of clinical complications^2^. HCC leads to 4.7% of newly-confirmed malignant diseases and 8.3% of tumor-related deaths globally^3^. The strategies towards HCC are diverse, ranging from radical resection, local ablation, transcatheter arterial chemoembolization (TACE), liver transplantation, and systemic therapies, among which resection is mostly utilized and commonly regarded as most effective^4^.

Centrally located HCC, which commonly exists in divergence of portal vein, junction of primary hepatic vein, inferior vena cava or less than 1 cm (cm) from posterior inferior vena cava trunk, mostly lied in Couinaud segment I, IV, V, VIII, or among convergence of core sections ^5,6^. Since centrally located HCC is neighbouring to dominant blood vessels and bile ducts, its clinical therapy is rather challenging and draws much attention globally, with the rate of relapse rising up to 90%, while the 5-year DFS only around 15–30%^7^. The probability of relapse would significantly escalate when margins of resection are narrower (< 1 cm) or even null, facilitating the diffusion of microscopic residual lesions via intrahepatic vessels^8^. Under this condition, it has been a critical and topical issue to conduct researches over combined therapies on the basis of surgical resection.

At present, surgical resection is considered as the core choice for resectable tumor mass of HCC patients, with adjuvant therapies performed based on pathological examinations postoperative. In preceding researches, the radiotherapy (RT) has been proved to be a safe and effective type of adjuvant therapy for centrally located HCC. Recently, it has been accessible for patients to receive accurate radiotherapy thanks to technical advances, like the appearance of intensity-modulated radiation therapy (IMRT), stereotactic body radiation therapy (SBRT) and three-dimensional conformal radiotherapy (3D-CRT)^9–11^. In this way, we explored the effect of adjuvant radiotherapy accompanied by surgical resection in order to solve the problem above.

As far as I know, there was no real-world research analyzing possible prognostic benefits of adjuvant radiotherapy following surgical resection for centrally located HCC before, and this is the aim for the study to bridge the gap.

## Materials and methods

### Selection of patients

Data for patients who underwent resection for HCC ranging from June 2015 to 2020 were gathered. People were recruited based on the including and excluding standards below. The content of inclusion criteria were as follows: (1) no less than 18 years old; (2) without extrahepatic metastasis; (3) centrally located tumor adhered to or within < 1 cm from the portal vein, hepatic vein, primary hepatic branch of the biliary system or retrohepatic inferior vena cava verified via imaging before surgery or intraoperative macroscopic test; (4) integral clinical and pathological information; (5) Child‒Pugh class A; and (6) ECOG 0 or 1. The excluded criteria were presented below: (1) non-HCC confirmed by pathological examination postoperative; (2) radiotherapy before tumor resection. Recruitment of all patients was decided by a multidisciplinary team (MDT) containing surgeons, physicians, radiologists, pathologists, etc., who were all covered in the determination regarding the treatment of patients.

### Treatment

#### Surgical treatment

We held a discussion in the form of MDT for every patient before surgery. The standardization of team. Initially, by performing exploratory laparotomy on the abdomen and pelvis, the condition of extrahepatic metastasis was examined, during which intraoperative ultrasound would be used to check the liver cancer if necessary. The region of resection was comprehensively decided by the general tumor and cirrhosis of liver. All patients underwent radical resection of HCC (R0 resection) to ensure that the postoperative pathological margin was tumor negative. During the operation, selective and dynamic region-specific vascular occlusion (SDRVO) technique was utilized to ascertain accurate liver resection individually^5^. Possible choices for HCC surgical resection contained anatomic hepatectomy and nonanatomical hepatectomy. The tumor was peeled off from the surface of biliary tract or large blood vessels via Cavitron Ultrasonic Surgical Aspirator (CUSA) without incision margins to refrain from cutting off primary vessels, when the tumor mass was adhered to critical tracts.

#### Adjuvant radiotherapy

Postoperative IMRT was applied to every patient in the LR + RT group, which was arranged and performed as depicted before^11^. In brief, tumor bed along with a 1 cm margin was considered as the clinical target volume (CTV). However, it was decided with a 1.5 cm margin surrounding the tumor bed when the tumor mass located next to main vessels. In the left–right and anterior–posterior directions, a 0.5-cm margin around the CTV was contained in the planning target volume (PTV), while in the cranial-caudal direction, a 1.0-cm margin was included. According to the PTV, the dose of RT was prescribed. Mainly dependent on dose constraints of specific organs, the prescribed dose for 95% of the PTV was arranged as 50–60 Gy among 25–30 fractions across 5–6 weeks.

#### Follow-up

Recurrence was defined in this way: fast in and out features revealed in the imaging of hepatocellular nodular (≥ 2 cm) or HCC verified via histological/cytological examinations. AFP levels, liver and kidney function examinations, regular blood check, abdominal magnetic resonance imaging (MRI), computed tomography (CT) scans and chest X-ray were routinely checked. Since operation, patients were rechecked each 3 months in the first 2 years, each 4–6 months before 5 years, followed by each 6–12 months since that. Generally, subjects would be evaluated if they undergo physical discomfort among follow-up, which was continued up to May 2022 for all of the subjects. This research got approval from the Ethics Committee of Cancer Hospital of Chinese Academy of Medical Science. Without interrupting process of diagnosis and therapy, this was a nonintervention cohort study, whose results would be published via statistical data of analysis and exclude information that could identify patients. On the basis of the Helsinki Declaration, we did not reveal related data of patients, and all participants were willing to participate in this study and gave their informed consent.

#### Treatment of recurrence

According to features of the tumor, liver function, overall status and decision from the patient, as well as suggestions from MDT, strategies including reoperation, hepatectomy, radiofrequency ablation (RFA), TACE, systemic therapy like immunotherapy or molecular targeted therapy were employed to deal with HCC relapse.

### Definition and analysis

The period from the data of operation to the time of relapse was referred to as disease-free survival (DFS). Aside from RT, we selected variables with possible influences on survival rate to calculate a propensity score^12^. We utilized the Clavien grading system to assess complications among hospitalizations, which were as follows: Grade I: intervention by drugs, endoscopy, radiotherapy and operation was not needed since operation, but physiotherapy, diuretics, electrolytes, antiemetics, antipyretics and antipyretics were permitted; Grade II: medicine except ones included in Grade I were required, containing parenteral nutrition and blood transfusion; Grade III: intervention by endoscopy, radiotherapy or operation was in need; Grade IV: life-threatening syndromes, like central nervous system (CNS) complications (cerebral hemorrhage and subarachnoid hemorrhage), and entry into the ICU were in requirement; and then Grade V: dead among hospitalization. We regarded occurrence of grade I or II syndromes to be mild level, while grade III, IV, and V to be severe. Clinical target volume (CTV) is the range of tumor focus and its possible infiltration; Planning target volume (PTV) include the CTV and the error range caused by positioning or movement, posture repeatability, and target volume movement.

### Statistical methods

Analysis was performed by steps below: (1) compare baseline data of two groups via standardized mean difference (SMD); (2) priori verification of confounders which might confound results (based on relations of confounders with results of interest or modifications in effect estimation of over 10%); (3) utilize Cox proportional-hazards regression models to evaluate correlation among exposed elements and prognosis, which includes crude and multi-variable calculation; (4) employment of three matching methods, covering Propensity-score Match (PSM), Covariate adjustment for Propensity Score (CAPS) and Inverse Probability Weighting (IPTW) to reduce the difference among groups and control confounding; (5) apply Kaplan‒Meier method to evaluate DFS, and log-rank test to calculate variance between groups; (6) research clinical benefits of radiotherapy upon early stage of relapse via subgroup analysis; (7) explore the potential of unsurveyed confounding among cohorts by counting E-value^13^. E-values quantify necessary magnitude of unsurveyed confounders which might deny calculated correlation between RT and DFS.

The 1:1 matching algorithm was utilized to produce matched cohorts between groups with a caliper of 0.02 set towards the scale of propensity score. And there was no replacement for 1:1 sampling. We compared all characteristics of patients which were contained in producing and distributing propensity scores via standardized mean difference (SMD), before and after matching propensity scores. We considered the threshold no more than 0.1 to be acceptable^14^.

Descriptive analyses were used to describe the demographic and clinical characteristics of participants. Categorical variables were described by frequency and percentage. Continuous variables that follow a normal distribution are described by the mean and standard deviation, while continuous variables that do not follow a normal distribution are described by the median and interquartile ranges. The analysis of statistics was conducted by R software, version 4.2.0 (http://www.R-project.org) and IBM SPSS Statistics 23.

### Ethical approval and consent to participate

This study was approved by the Ethics Committee of Cancer Hospital of Chinese Academy of Medical Science. All methods were carried out according to the Helsinki Declaration. The relevant data for the patients remained confidential, and all participants were willing to participate in this study and gave their informed consent.

## Results

### Patients

**Figure 1:**
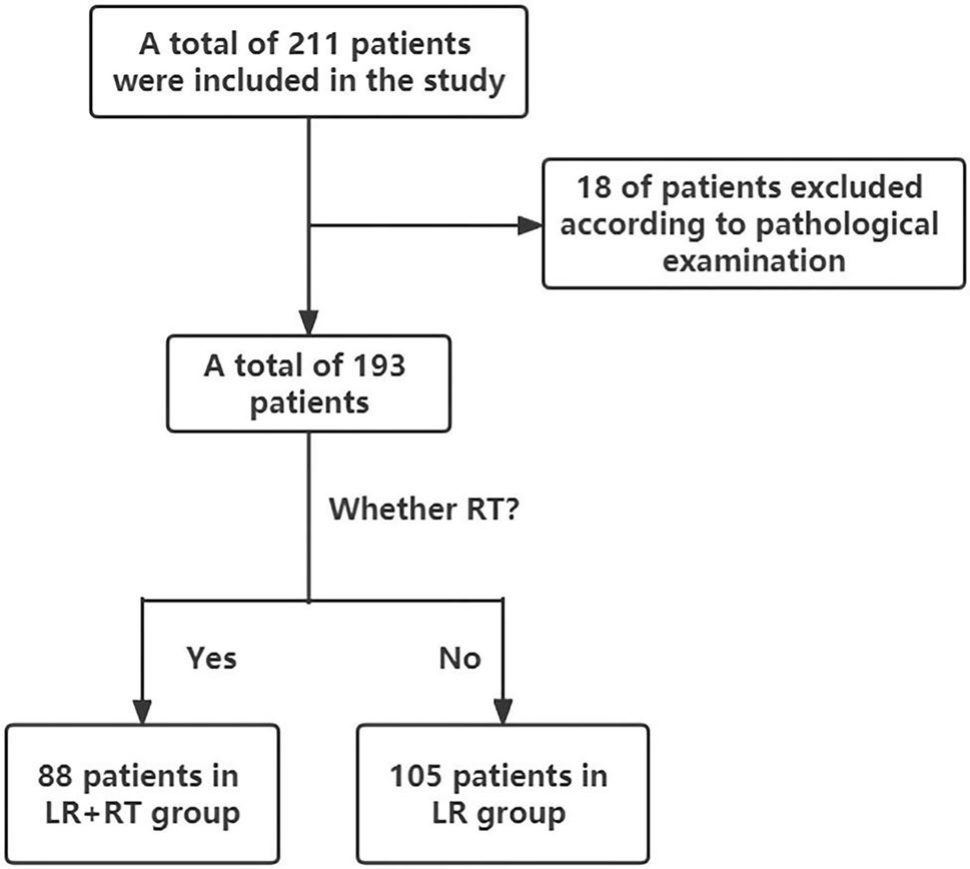
Flow chart for patient screening.

We recruited 211 patients in total based on the inclusion criteria; and 18 of them were excluded due to the exclusion criteria. Then, 193 patients were finally chose in all. Based on whether RT was employed, we allocated the patients into two groups, surgical resection along with adjuvant radiotherapy (LR + RT, 88 patients) or mere liver resection (LR, 105 patients) groups. The rates for 1-, 3-, and 5-year DFS were 98%, 85% and 74% for patients in LR + RT cohort, while 76%, 55%, and 44% respectively in the LR group. The flow chat for screening of patients is given in Fig. 1. In Table 1, we shown baseline demographics and clinicopathological characteristics of patients. Before matching, the variance between groups was notable (SMD > 0.1) according to the standardized mean difference.

**Table 1:** Comparisons of Baseline demographics and clinicopathological characteristics in patients undergoing LR + RT or LR alone before and after propensity score matching analysis.

Characteristic	Before matching			After matching		
	LR + RT (n = 88)	LR (n = 105)	SMD^a^	LR + RT (n = 70)	LR (n = 70)	SMD^a^
Age (years)			0.17			0
≤ 60	62 (70.5%)	81 (77.9%)		55 (78.6%)	55 (78.6%)	
> 60	26 (29.5%)	23 (22.1%)		15 (21.4%)	15 (21.4%)	
Sex			0.16			0.04
Male	72 (81.8%)	92 (87.6%)		60 (85.7%)	61 (87.1%)	
Female	16 (18.2%)	13 (12.4%)		10 (14.3%)	9 (12.9%)	
Alcoholism			0.05			0
Yes	29 (33.0%)	32 (30.5%)		22 (31.4%)	22 (31.4%)	
No	59 (67.0%)	73 (69.5%)		48 (68.6%)	48 (68.6%)	
Smoke			0.17			0.06
Yes	37 (42.1%)	53 (50.5%)		30 (42.9%)	32 (45.7%)	
No	51 (57.9%)	52 (49.2%)		40 (57.1%)	38 (54.3%)	
Diabetes			0.10			0.04
Yes	14 (15.9%)	13 (12.4%)		9 (12.9%)	10 (14.3%)	
No	74 (84.1%)	92 (87.6%)		61 (87.1%)	60 (85.7%)	
Body mass index (BMI)			0.20			0.06
≤ 18.5	0 (0%)	2 (1.9%)		0 (0%)	0 (0%)	
> 18.5, ≤ 24	36 (40.9%)	42 (40.0%)		30 (42.9%)	28 (40.0%)	
> 24	52 (59.1%)	61 (58.1%)		40 (57.1%)	42 (60.0%)	
HBV			0.18			0.03
Yes	59 (67.1%)	79 (75.2%)		50 (71.4%)	49 (70.0%)	
No	29 (32.9%)	26 (24.8%)		20 (28.6%)	21 (30.0%)	
HCV			0.20			0
Yes	12 (13.6%)	8 (7.6%)		8 (11.4%)	8 (11.4%)	
No	76 (86.4%)	97 (92.4%)		62 (88.6%)	62 (88.6%)	
Preoperative liver function						
ALT (U/L)	23.0 (17.8–35.0)	32.0 (23.0–41.0)	0.29	25.0 (19.0–36.8)	27.5 (23.0–35.8)	0.04
AST (U/L)	24.0 (20.0–31.0)	31.0 (25.0–43.0)	0.38	26.0 (21.0–31.8)	28.0 (23.0–33.0)	0.07
TBIL (μmol/L)	13.0 ± 4.4	13.4 ± 5.6	0.08	13.0 ± 4.6	13.2 ± 5.3	0.03
Liver tumor						
Tumor size (cm)	5.1 ± 2.4	5.8 ± 2.7	0.27	5.3 ± 2.6	5.2 ± 2.3	0.04
MVI			0.18			0.03
Yes	72(81.8%)	78(74.3%)		55 (78.6%)	54 (77.1%)	
No	16(18.2%)	27(25.7%)		15 (21.4%)	16 (22.9%)	
Number			0.09			0.09
Single	78 (88.6%)	90 (85.7%)		62 (88.6%)	64 (91.4%)	
Multiple	10 (11.4%)	15 (14.3%)		8 (11.4%)	6 (8.6%)	
AFP			0.08			0.13
≤ 400 ng/mL	65(73.9%)	74(70.5%)		49(70.0%)	53(75.7%)	
> 400 ng/mL	23(26.1%)	31(29.5%)		21(30.0%)	17(24.3%)	
Intraoperative bleeding (mL)	200.0 (100.0–600.0)	400.0 (200.0–600.0)	0.17	200.0 (100.0–600.0)	350.0 (125.0–600.0)	0.09
Complication			0.01			0
Mild	84 (95.5%)	100 (95.2%)		66 (94.3%)	66 (94.3%)	
Severe	4 (4.5%)	5 (4.8%)		4 (5.7%)	4 (5.7%)	

Variables are expressed as the mean ± SD(median with range), median (Q1-Q3) or N(%) (number with percentages), unless otherwise indicated. RT, adjuvant radiotherapy; LR, liver resection.

^a^Standardized mean difference (SMD): Standardized differences of > 0.1 represent meaningful differences in covariates between groups.

### Cox regression

**Figure 2:**
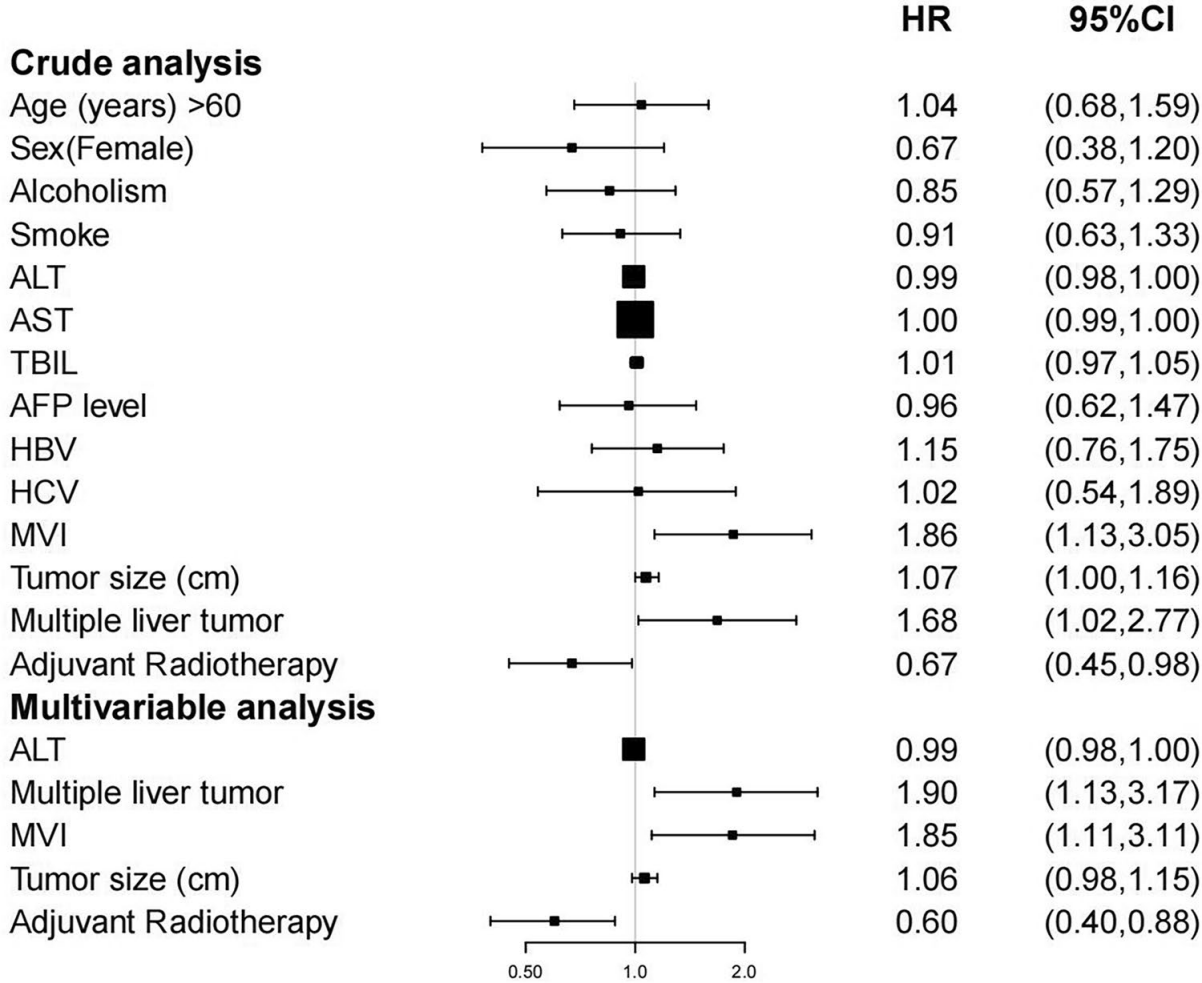
Cox proportional hazards regression in DFS.

As it is revealed through the forest plot in Fig. 2 via crude analysis, significant correlation was existent between LR + RT and enhanced DFS (HR 0.67, 95% CI [0.45, 0.98], Table 2, Fig. 2). Afterwards, confounding elements were filtered out based on the strategies above, containing ALT, MVI, number and size of tumor. We covered the aforementioned confounding elements in the multiple regression equation for adjustment. And RT was disclosed to be an independent factor of prognosis for centrally located HCC via multivariate Cox analysis (HR 0.60, 95% CI [0.40, 0.88], Table 2, Fig. 2).

**Table 2:** Associations between LR + RT and LR alone group in the Crude Analysis, multivariable analysis and propensity-score analysis.

Analysis	Hazard ratio(95% CI)
Crude analysis	0.67 (0.45, 0.98)
Multivariable analysis^1^	0.60 (0.40, 0.88)
Propensity-score analyses	
With matching^2^	0.60 (0.38, 0.94)
Adjusted for propensity score^3^	0.60 (0.38, 0.94)
With inverse probability weighting^4^	0.63 (0.40, 0.98)

^1^Shown is the hazard ratio from the multivariable Cox proportional-hazards model, with stratification according to adjuvant radiotherapy, and with additional adjustment for confounders tumor number, tumor size, MVI and ALT level. The analysis included all patients.

^2^Shown is the hazard ratio from a multivariable Cox proportional-hazards model with the same strata and covariates with matching according to the propensity score (Confounders or Covariates included sex, age, smoke, alcohol, ALT level, AST level, TBIL level, HBV, HCV, tumor number, tumor size, MVI and alpha-fetoprotein; caliper 0.02). The analysis included 140 patients (70 who received LR + RT and 70 who received LR alone).

^3^Shown is the hazard ratio from a Cox proportional-hazards model with the same strata and covariates, with additional adjustment for the propensity score. The analysis included 140 patients (70 who received LR + RT and 70 who received LR alone).

^4^Shown is the primary analysis with a hazard ratio from the multivariable Cox proportional-hazards model with the same strata and covariates with inverse probability weighting according to the propensity score. The analysis included all the patients.

### Propensity score analyses

Initially, we utilized propensity-score matching (PSM) method to decrease the discrepancy among groups due to imbalanced baseline information. We covered baseline information and confounders, including sex (Male or Female), age (≤ 60 or > 60), smoking (Yes or NO), alcohol consumption (Yes or NO), ALT value, AST value, TBIL value, HBV (Yes or NO), HCV (Yes or NO), tumor number (Single or Multiple), tumor size, and MVI (Yes or NO) as matching factors. 70 patients in total were successfully matched. As revealed in Table 1, there was no significant difference (SMD < 0.1) in baseline characteristics of patients among groups after matching. However, the association between LR + RT and prolonged DFS was significant after PSM (HR 0.60, 95% CI [0.38, 0.49], Table 2). Then, covariate adjustment applying propensity score (CAPS)^15^ was utilized to regulate confounding. After CAPS, the association between LR + RT and prolonged DFS was notable (HR 0.60, 95% CI [0.38, 0.49], Table 2). At last, IPTW was adopted to count the stabilized inverse-probability weight by analyzing predicted probabilities by previous propensity-score model^16^. After IPTW, we revealed the existence of significant correlation between LR + RT and extended DFS (HR 0.63, 95% CI [0.40, 0.98], Table 2). Therefore, through statistical method, notably prolonged DFS in the LR + RT group was statistically verified compared with the LR group on the basis of real-world data.

### Survival analysis

**Figure 3:**
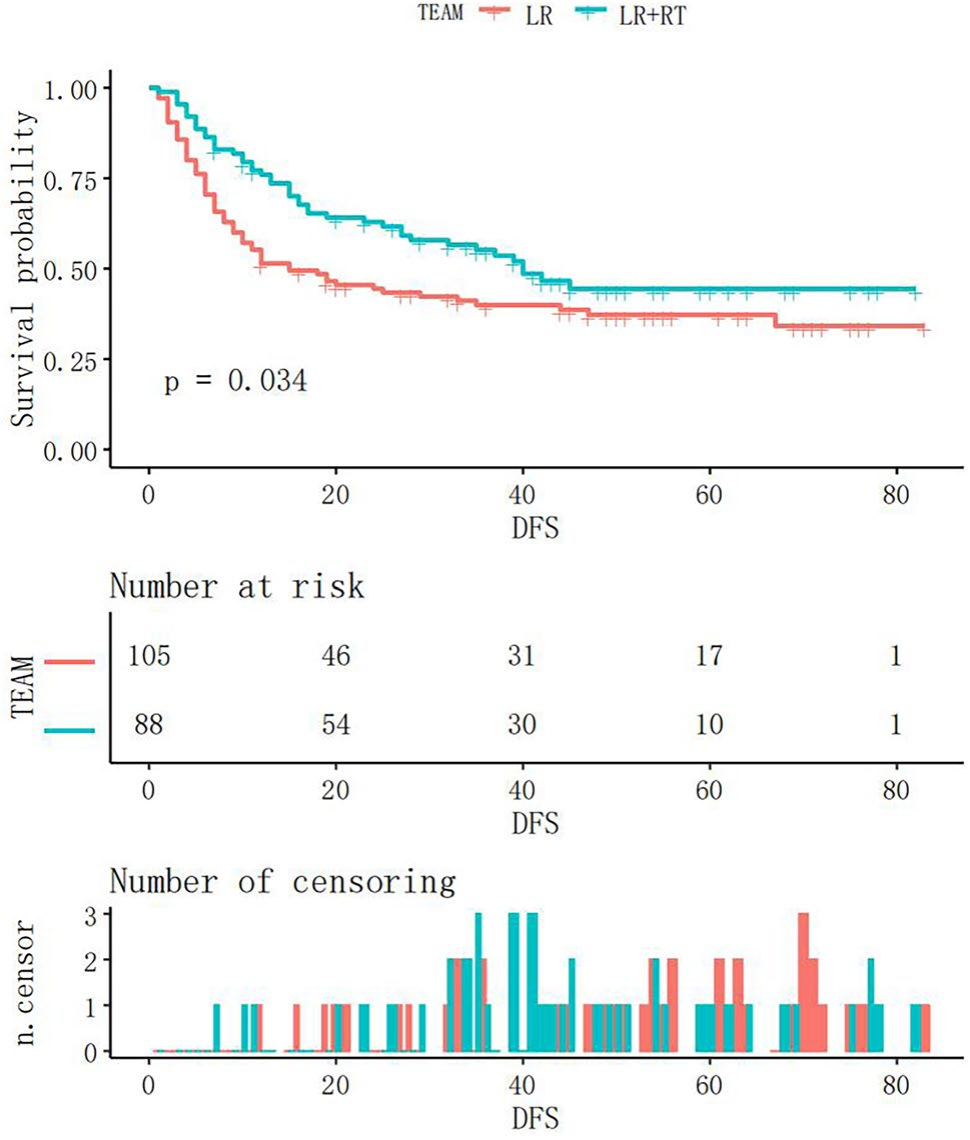
Kaplan‒Meier curve of DFS before matching in the LR + RT and LR groups. RT, adjuvant radiotherapy; LR, liver resection.

**Figure 4:**
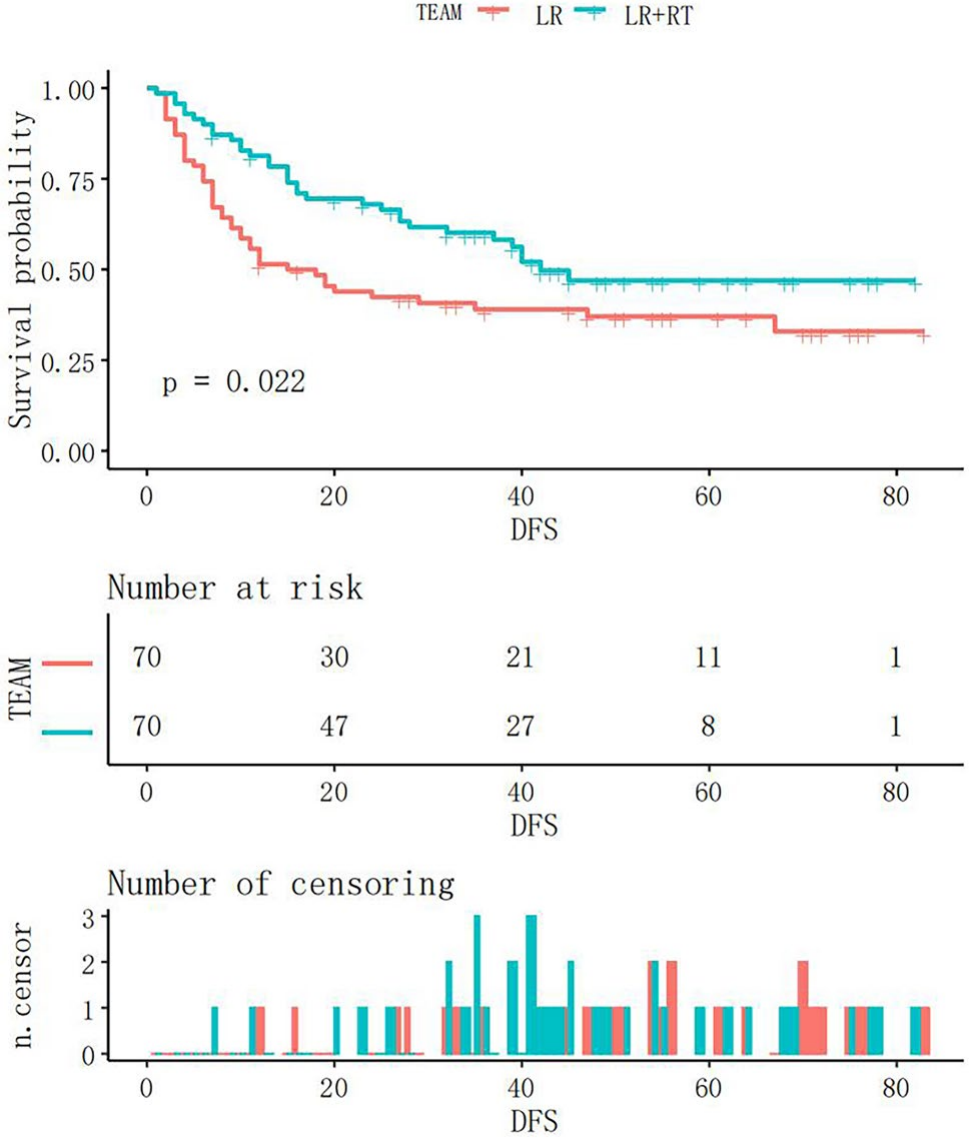
Kaplan‒Meier curve of DFS after matching in the LR + RT and LR groups. RT, adjuvant radiotherapy; LR, liver resection.

Before matching analysis, the Kaplan‒Meier curve was given in Fig. 3 with significance between groups (*p* value = 0.034). Afterwards, by matching 70 patients at 1:1, there was no apparent difference on baseline data. And Fig. 4 presented the Kaplan‒Meier curves of DFS after matching analysis, in which DFS was enhanced and remarkably distinct in the LR + RT group compared with that of the LR group (*p* value = 0.022).

### Recurrence pattern and subgroup analysis

After matching analysis, we encompassed 140 patients in all in the research; 77 of them developed relapse post-operation, involving 33 in the LR + RT group, while 44 in the LR group respectively. Moreover, the occurrence of intrahepatic relapse and extrahepatic metastasis was separately 31 and 2 for LR + RT group, while 41 and 3 for LR group. As the primary determinant of prognosis post hepatectomy, high rate of relapse would be separated into early and late stage of recurrence^17,18^, among which the clinical prognosis of patients experiencing early relapse has been widely reported to be worse than those with late stage of relapse in large quantities of researches^19–21^. Currently, 12 or 24 months are widely regarded to be proper time points for early stage of relapse by most scholars^17,22–24^. In this way, we performed subgroup calculation to further explore the influence of RT upon early stage relapse, disclosing that RT would decrease the occurrence of relapse whatever the time point for early relapse was set up at 12 or 24 months (*p* value = 0.001, *p* value = 0.002, respectively. Table 3).

**Table 3:** Comparisons of early recurrence in patients undergoing LR + RT or LR.

	LR + RT (n = 70)	LR (n = 70)	Standardized difference	*p*-value
Early recurrence (12 months)			0.70	0.001
Yes	13	35		
No	57	35		
Early recurrence (24 months*)*			0.53	0.002
Yes	24	42		
No	46	28		

RT, adjuvant radiotherapy; LR, liver resection.

### Sensitivity analysis

An E-value was produced to evaluate the sensitivity of unsurveyed confounding. The initial results were robust unless there existed unsurveyed confounder, whose HR was higher than 2.20. And in this study, the analysis of E-value indicated robustness to unsurveyed confounder.

### Complications and safety

When it comes to safety, all of 70 patients across both groups succeeded in undergoing operation and and all underwent R0 resection. There were no significant differences upon intraoperative bleeding and duration of operation in two cohorts (*p* value = 0.614, *p* value = 0.125, separately). Moreover, people across two groups acquired proper operative strategies without occurrence of perioperative death. Mild and severe complications after matching analysis were not significantly variant (66 mild complications and 4 severe complications in both groups, *p* value > 0.05).

## Discussion

In the conventional concept of centrally located HCC, it is regarded as the tumor mass located in IV, V and VIII Couinaud segments of the liver^25^. Since this concept fails to emphasize the association between tumor and peripheral structures, including primary bile duct and blood vessel, it is of poor guidance for the surgery. We put forward a revised version of definition, in which central located HCC is referred to liver tumor adhered to or within 1 cm from the hepatic vein, portal vein, primary hepatic branches of biliary system or retrohepatic inferior vena cava verified by imaging before surgery, macroscopic examination intraoperative, or pathological results post operation, commonly lied in Couinaud segment I, IV, V, and VIII, or located at the convergence of core sections^6,26^. Owing to its extreme proximity from the vein, the centrally located HCC is characterized by demanding surgery, poor rates of radical resection, diverse postoperative complications and high rates of relapse. Generally, understanding and exploration towards its therapeutic strategies symbolize the gradual progress of clinical medicine treatment towards liver cancer. It is rather challenging to figure out proper methods to safely get rid of centrally located HCC and upregulate OS of HCC patients post operation. To solve this problem, our surgical group has carried on long-term relevant researches upon combined therapy towards centrally located HCC on the basis of resection^9,11,26–28^.

This is the first real-world research analyzing promising advantages of liver resection integrated with adjuvant radiotherapy towards centrally located HCC, whose consequences reveal that comprehensive therapy possesses remarkable effects and notably upgrades clinical prognosis.

As a critical type of therapeutic choice, radiotherapy has been broadly utilized upon clinical remedy for various types of malignant tumor. Since the rapid technical advances of accurate radiotherapy which enable the arrival of high-dose radiation rays to the targeting region, radioactive injuries towards the surrounding normal liver tissues and other organs are effectively cut down. According to the demonstration from other related researches, clinical prognosis of HCC patients might be enhanced post radiotherapy. And corresponding to our phase II clinical trial and other research studies^9–11,26,29,30^, adjuvant radiotherapy is efficient, well tolerated, and potential for patients with centrally located HCC. Compared with previous researches, this research is superior from the following aspects. Initially, we made sure that all of patients that we recruited had access to clinical treatment and management of high quality from the same team of doctors in the hospital. Second, the statistical means we employed to cut down selection bias of patients added to the authenticity and reliability of our results. Moreover, the comparably long time of follow-up facilitated the reflection of long-term prognosis and functioned better upon clinical guidance.

Based on the consensus of guideline, a conserved minimized surrogate threshold effect of HR ≤ 0.6 towards DFS would be capable of suggesting notable rising upon overall survival (OS)^31^. And since our outcomes met this standard (Table 2), it is reasonable to assume the capacity of radiotherapy to prolong OS of patients with centrally located HCC post operation. In multivariate analysis, RT was confirmed to be an independent prognostic factor, while multiple liver tumor and MVI were confirmed to be independent risk factors. Most studies believe that MVI is a high-risk factor for intrahepatic metastasis and postoperative recurrence^20,22^, which will eventually lead to the occurrence of multiple tumors in the liver, which is consistent with our results. In addition, research has shown that AFP value is a risk factor for the OS or DFS of patients with HCC after surgery^32^. For these high-risk patients, in addition to postoperative RT, a previous study showed that liver transplantation is also one of the effective treatment methods^32^. Clinically, the appropriate treatment should be selected according to the actual situation of patients.

In terms of the extended survival time, there exists several possible reasons. To begin with, RT decreases the potential harm caused by narrow margin resection, which has been verified that, resection with wide-margin results in prolonged OS compared with that of narrow-margin ones (< 1 cm)^33,34^. Furthermore, it is interesting to note that RT effectively decrease the rate of relapse in the early stage, which might partly due to its destruction towards minimal residual disease (MRD)^35,36^. Decline of relapse rate facilitates the enhanced OS. Nevertheless, limitations still exist in this research, for which our study was still a retrospective research, and further randomized controlled studies are still in need to verify our consequences.

## Conclusion

Combination of surgical resection with adjuvant radiotherapy is safe and effective for remedy of patients with centrally located HCC, which would clearly enhance clinical outcomes and decline the occurrence of relapse in the early stage.

## Data Availability

All data related to this study are included in this paper. Details are available from the corresponding author on reasonable request.

## Abbreviations

HCC: Hepatocellular carcinoma
RT: Adjuvant radiotherapy
LR: Liver resection
SMD: Standardized mean difference
PLC: Primary liver cancer
TACE: Transcatheter arterial chemoembolization
RT: Radiotherapy
SDRVO: Selective and dynamic region-specific vascular occlusion
OS: Overall survival
DFS: Disease-free survival
PSM: Propensity-score match
CAPS: Covariate adjustment for Propensity Score
IPTW: Inverse probability weighting
